# Stimulated photon emission and two-photon Raman scattering in a coupled-cavity QED system

**DOI:** 10.1038/srep20991

**Published:** 2016-02-15

**Authors:** C. Li, Z. Song

**Affiliations:** 1 School of Physics, Nankai University, Tianjin 300071, China

## Abstract

We study the scattering problem of photon and polariton in a one-dimensional coupled-cavity system. Analytical approximate analysis and numerical simulation show that a photon can stimulate the photon emission from a polariton through polariton-photon collisions. This observation opens the possibility of photon-stimulated transition from insulating to radiative phase in a coupled-cavity QED system. Inversely, we also find that a polariton can be generated by a two-photon Raman scattering process. This paves the way towards single photon storage by the aid of atom-cavity interaction.

A coupled-cavity QED system provides a promising platform to study novel quantum phenomena, since it combines two or more distinct quantum components, exhibiting features not seen in these individual systems. The discrete spatial mode of the photon in a coupled-cavity array and its nonlinear coupling to atom make the possible applications both in quantum information processing^1^ and quantum simulation^2^. The seminal papers^3^,^4^,^5^ proposed the use of the system to create strongly correlated many-body models. It has predicted the quantum phase transition from Mott insulator phase to superfluid phase^5^,^6^. This scenario is constructed under the assumption that there is no extra photon leaking into the system. The stability of an insulating phase bases on the fact that the polariton states in a cavity QED system are eigenstates, i.e., spontaneous photon emission is forbidden. This situation may change if a photon can stimulate the photon emission from a polariton. In contrast with quantum phase transition induced by varying system parameters, such as atom-cavity coupling strength, stimulated photon emission from polaritons can also trigger the transition between insulating and radiative phases. It is interesting and important to investigate the photon-photon and photon-polariton scattering processes. Many efforts related to few-body dynamics mainly focused on multi-photon transports through coupled-cavity QED systems^7^,^8^,^9^,^10^,^11^,^12^,^13^,^14^,^15^,^16^,^17^,^18^,^19^, while a few works dealt with the formation of bound state^20^,^21^,^22^. So far, what happens when a photon collides with a polariton is still an open question.

In this paper, we study the scattering problem of an incident photon by a polariton in a one-dimensional coupled-cavity QED system. Analytical approximate analysis and numerical simulation reveal several dynamical features. We find that a photon can stimulate the photon emission from a polariton, which induces the amplification of the photon population in a multi-polariton system. After a chain reaction, incident photons can stimulate the transition from insulating to radiative phases in the system with low doped cavity density. We also investigate the inverse process of stimulated photon emission from a polariton. We will show that a polariton can be generated by a two-photon Raman scattering process, which has been studied for the atoms found in nature^23^,^24^,^25^. Moreover, it has been shown that an atom-cavity system can behave as a quantum switch for the coherent transport of a single photon^26^. Considering a two-excitation problem, we find that a single-photon transmission through a quantum switch is affected significantly by a polariton that resides at it.

This paper is organized as follows. At first, we present the model and single-excitation polaritonic states. Then, we propose an effective Hamiltonian to analyze the possibility of photon emission from two aspects. Numerical simulations for two-particle collision processes are showed later. Finally, we give a summary and discussion.

## Results

### Model and polariton

We consider a one-dimensional coupled-cavity system with a two-level atom, which is embedded in the center of cavity array. The Hamiltonian can be written as


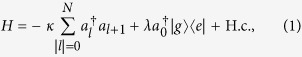


where *λ* represents atom-cavity coupling strength and *κ* is the photon hopping strength for the tunneling between adjacent cavities. Here, 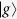 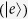 denotes the ground (excited) state of the qubit with 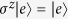 and 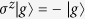, *a*_*l*_ 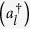 annihilates (creates) a photon at the *l*th cavity. Obviously the total excitation number, 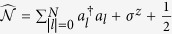, is a conserved quantity for the Hamiltonian *H*, i.e., 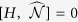.

The coupled-cavity array can be considered as a one-dimensional waveguide, while the two-level atom can act as a quantum switch to control the single-photon transmission^26^. To demonstrate this point, we rewrite the Hamiltonian in the form


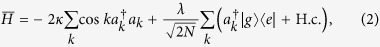


where


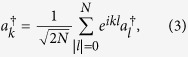



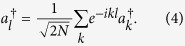


It indicates that the atom couples to photons of all modes 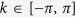. In the 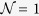 subspace, atom can be regarded as a stationary scattering center. All the dynamics can be treated in the context of single-particle scattering method, which has been well studied^26^.

A comprehensive understanding for the dynamics involving the sector with 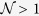 is necessary to both theoretical explorations and practical applications. Intuitively, the state of the atom 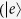 or 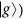 should affect the interaction between the atom and a photon. In experiments, the practical processes may concern two or more photons, which obviously affect on the function of the quantum switch. On the other hand, the stability of an insulating phase may be spoiled by the background photons from environment. In this paper, we study the scattering problem in the 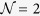 sector, focusing on the effect of the nonlinearity arising from the atom. The investigation has two aspects: First, we study the photon scattering from a polariton. Secondly, we consider the collision of two photons under the atom-cavity nonlinear interaction.

We start our investigation with the solution of single-particle bound and scattering states. In the invariant subspace with 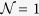, exact solution shows that there are two bound states, termed as single-excitation polaritonic states, being the mixture of photonic and atomic excitations. From the Method, these polaritonic states are obtained by Bethe Ansatz method as the form


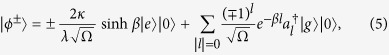


where the normalization factor is


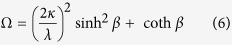


and


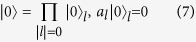


The corresponding energy is


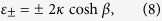


where the positive number *β* determines the extension of bound states around the doped cavity, obeys the equation


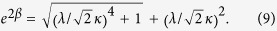


We can see that *β* has nonzero solutions for nonzero *λ*, indicating the existence of nontrivial bound states.

On the other hand, the derivation in Method shows that the solution of scattering states 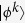 with energy 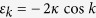 has the form


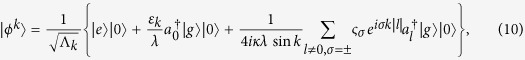


where Λ_*k*_ is the normalization factor and


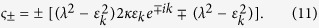


**Figure 1:**
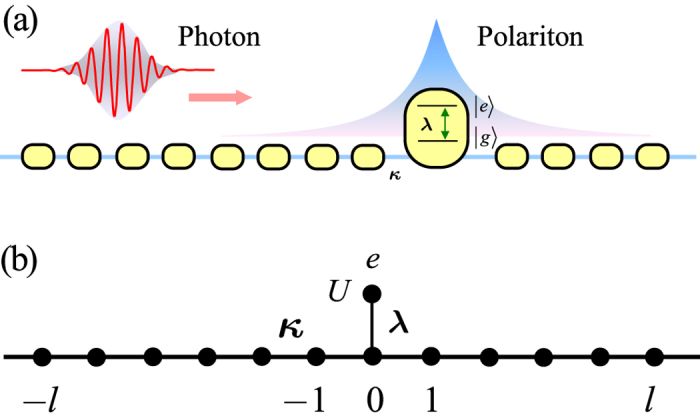
(**a**) Schematic configuration for the coherent collision of polariton and photon. An array of coupled single-mode cavities, where the central cavity is coupled to a two-level atom. Initially a polariton is located at the center, while a photon wave packet is moving from the left to collide with the polariton. (**b**) Schematic illustration for the equivalent description of the hybrid system. The excited state of the atom can be treated as a side-coupling site with infinite on-site repulsion.

We can see that a polariton is a local eigen state of the system, which is stable and cannot emit a photon in the subspace. The aim of this work is considering the effects of photon-photon and photon-polariton collisions. Our strategy is sketched in Fig. 1(a). In the invariant subspace with 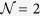, a two-excitation state can be a direct product of a local photon and a polariton states, which are well separated in real space. As long as time evolution, two local particles are overlapped. The nonlinear effect induces the interaction between the photon and polariton. After a relaxation time, the free photons spread out from the central cavity, only the polaritons are left, being stationary at the center. In the case of the ultimate polaritonic probability being less than 1, (or the escaped photon number larger than 1) we can conclude that the stimulated photon emission occurs during the process. We will show that this behavior becomes crucial when we study the stability of a macroscopic insulating phase, and the efficiency of a quantum switch in a waveguide. In the following section, we will analyze the possibility of photon emission from two aspects.

## Effective description

In this section, we present an analytical analysis on the effects of photon-photon and photon-polariton collisions. This will be based on an effective description of the original Hamiltonian *H* or 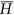. We extend the Hilbert space by introducing the auxiliary photon state 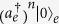, where 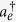 is the creation operator of a photon at site *e* and 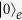 is the corresponding vacuum state. The qubit state 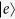 is replaced by 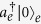. We rewrite the original Hamiltonians *H* and 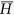 as the Hubbard models


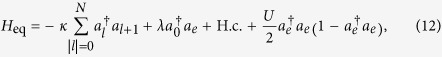


and


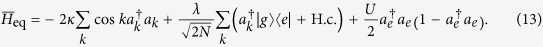


We note that the state 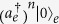 with *n* > 1 will be ruled out as 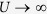, Hamiltonians *H*_eq_ and 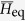 being equivalent to *H* and 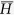, respectively. Correspondingly, we have 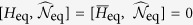 by defining 
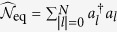
 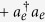. We will see that this equivalence can be true for a large magnitude *U* ~ 10. Next, we will perform our analysis from two aspects: *k* space and real space.

## Coupled equations in *k* space

First of all, we would like to point out that the eigenstates of the Hamiltionians *H* and 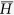 in Eqs (1) and (2) are still the eigenstates of *H*_eq_ and 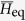 by taking 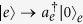. Now we consider the case in two-particle subspace. The basis set for two-particle invariant subspace can be constructed by the single-particle eigen states 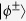 and 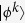. We concern the complete basis set with even parity, which can be classified into four groups


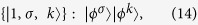



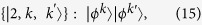



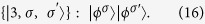


where *σ* = ±. We note that state 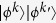 is automatically the eigenstate of *H* with eigen energy 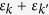. And states 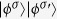 will be ruled out as *U* → ∞. Then basis sets 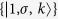 and 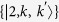 can further construct an invariant subspace approximately. In this sense, the solution of the Schrodinger equation


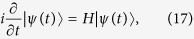


has the form


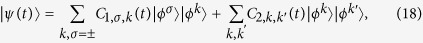


where coefficients 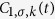 and 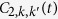 describe the two-particle dynamics and satisfy the coupled differential equations


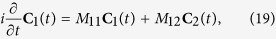



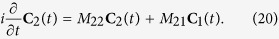


Here the column vectors 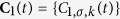 and 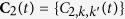, and the matrix


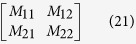


is a matrix representation of *H* on the basis set 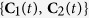. Although we cannot get an analytical solution of 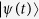, we can conclude that the nontrivial solution 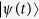 should predict the following relations in principle. We can always have nonzero **C**_2_(*t*) from initial condition **C**_1_(0) ≠ 0 but **C**_2_(0) = 0, i.e.,


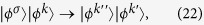


and inversely, nonzero **C**_1_(*t*) from initial condition **C**_2_(0) ≠ 0 but **C**_1_(0) = 0, i.e.,


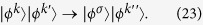


**Figure 2:**
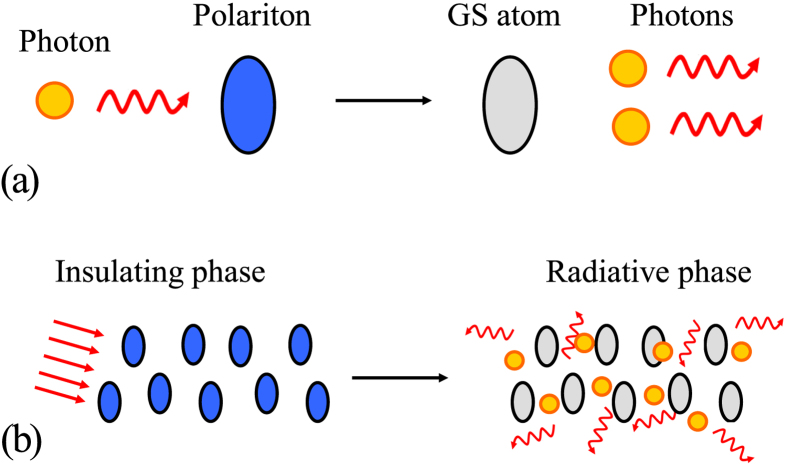
Polariton-photon transition in a coupled-cavity array coupled to a two-level atomic system. (**a**) When the collision between a photon and polariton occurs, the total photon probability cannot be preserved. The gain of photons indicates the stimulated photon emission. The blue (gray) color represents the polaritonic (atomic ground) state. (**b**) The insulating-radiative phase transition. A multi-polaritonic insulating state can collapse to a radiative state by an external field radiation.

**Figure 3:**
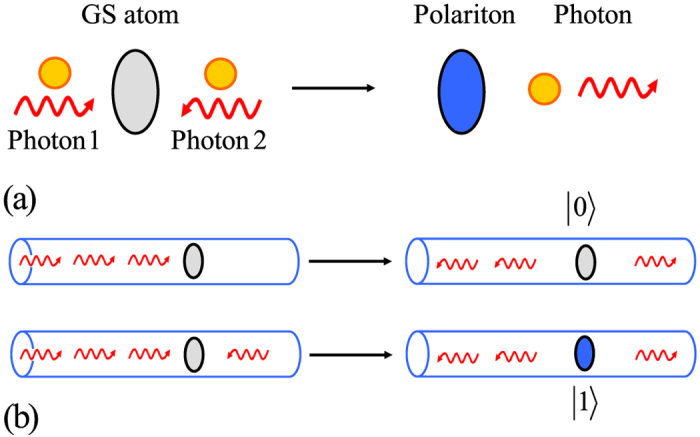
The two-photon Raman transition in a coupled-cavity array coupled to a two-level atomic system. (**a**) When the collision between two photons from opposite directions occurs at the cavity with an atom, the total photon probability cannot be preserved. The loss of photons indicates the two-photon Raman transition. The blue (gray) color represents the polaritonic (atomic ground) state. (**b**) Single-photon storage by the aid of single photons train from the opposite side. Any single photons cannot be stored in the atom when they transmit unidirectionally. It can be achieved by an incident single photons from the opposite side.

The former corresponds to the stimulated photon emission of the polariton, while the latter corresponds to the polariton state generation by a two-photon Raman scattering. The two processes are schematically illustrated in Figs 2(a) and 3(a).

## Effective photon blockade

In this section, we will demonstrate the process in Eq. (22) from an alternative way. One can consider the collision between an incident photon and an initial bound state around the site *e* in the system *H*_eq_. The obtained result should be close to that of the *H* system. In this context, the photon-photon collision only occurs at site *e*. Then the impact of the incident photon on the bound photon can be approximately regarded as a kicked potential on the *e*th site. In the following, we will investigate the effect the potential works on the dynamics of the bound photon.

We reduce the two-particle system of *H*_eq_ to a single-particle system with the effective time-dependent Hamiltonian,


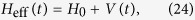



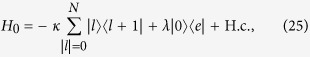



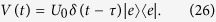


where *U*_0_ is the strength of the scattering and 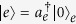, 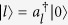 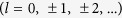 denotes the single-photon state. The initial state is one of the bound states


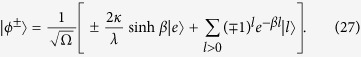


After the impact of the kicked potential, 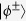 should probably jump to the scattering states 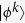. In the following, we demonstrate this point based on time-dependent perturbation theory.

For small *U*_0_, the transition probability amplitude from the initial state 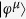 at *t* = 0 to 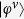 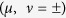 at *t* > *τ* can express as


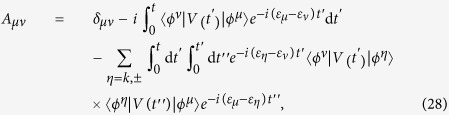


up to second order according to the time-dependent perturbation theory. Using the identity


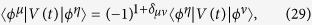


and the completeness condition


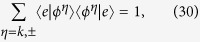


we get the transition probability between two bound states


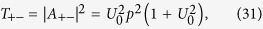



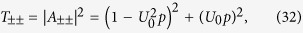


where


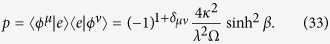


The crucial conclusion is that the transition probability from the bound state to the scattering state is


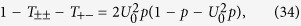


which is always positive for small nonzero *U*_0_. This indicates that the collision between a photon and a polariton can induce the photon emission from the polariton.

We employ the numerical simulation for verification and demonstration of our analysis. We compute the time evolution of an initial bound state by taking a rectangular approximation to a delta function.


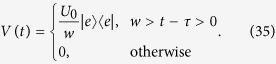


**Figure 4:**
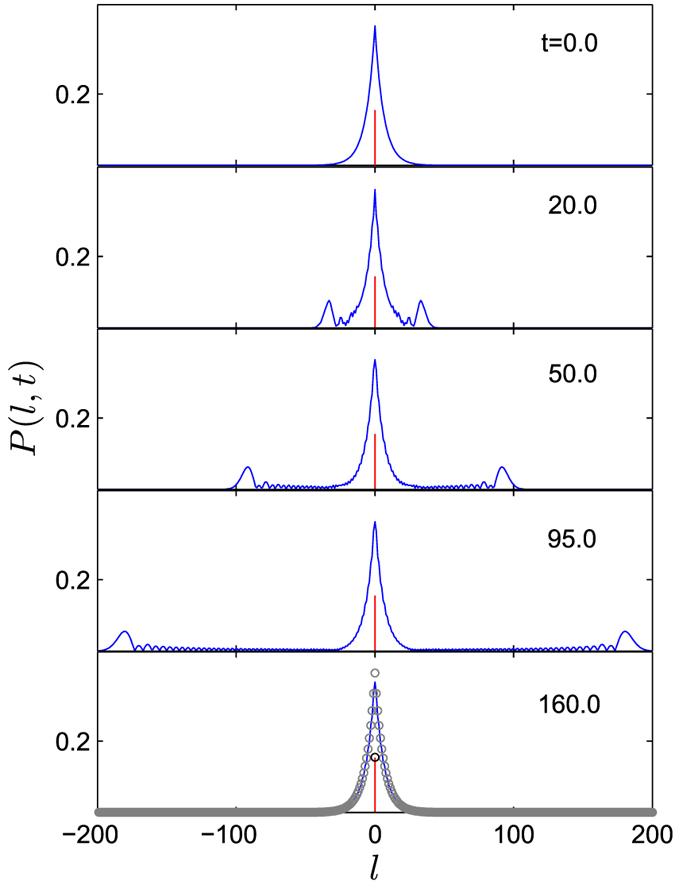
Time evolution of the initial bound state 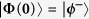 in Eq. (27) driven by the rectangular-pulsed potential. The magnitude distributions of the evolved wave function 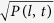 for several instants are obtained as converging results for *λ* = 0.8, *U*_0_ = 2, and 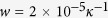. The red line indicates the probability of the *e*th site. The circle(black) represents the initial profile of the bound state at site *e* and the circles (gray) represents the initial profile of the bound state as comparison to the profile of the final state. It shows that there is a particle probability spreading out from the center to the infinity, and the final bound state has almost the same shape as the initial one but less probability. This indicates that a kicked potential can induce the transition from the bound states to the scattering states.

For fixed *U*_0_, we carry the calculation for different values of *w*. It is found that the result becomes stable as *w* decreases. The convergent data are adopted as an approximate numerical result. The evolution of an initial bound state under the central potential pulse is computed as well. The magnitude distribution of the evolved wave function 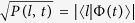 is plotted in Fig. 4. Here the propose of using 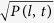 rather than the probability *P*(*l, t*) is to highlight the escaping wave packets from the center. We can see that there are two sub-wave packets propagating to the leftmost and rightmost, and the amplitude of the central bound state is reduced after this process. It can be predicted that the bound-state probability will keep decreasing by the successive pulses potential.

The result of this section cannot be regarded as sufficient proof of the occurrence of the stimulated photon emission from a polariton. Nevertheless, it shows that there is a high possibility that such a process can happen. In the following section, we will investigate this phenomenon by numerical simulation.

## Numerical simulation

In principle, one can explore the problem by solving the coupled equations (19) numerically. The truncation approximation is necessary since a numerous number of equations are involved. However, we can take an alternative way for truncation approximation, which is more efficiency for a discrete system. We can solve the Schrodinger Eq. (17) in finite real space by computing the time evolution of the initial state


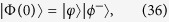


where 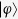 denotes local photonic state which is separated from polariton 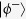 in real space. The following analysis is also available for the state 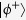. At time *t*, the evolved state is


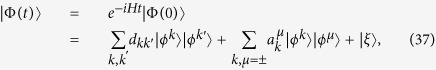


where 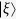 denotes two-excitation polaritonic state. We consider the local photonic state 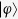 as a Gaussian wave packet with momentum *k*_0_ and initial center *N*_*A*_, which has the form


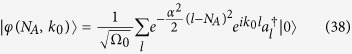


where 
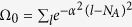
 is the normalization factor and the half-width of the wave packet is 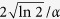. We take 
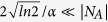
 to ensure the two particles being well separated initially. The evolved wave function 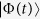 is computed by exact numerical diagonalization.

**Figure 5:**
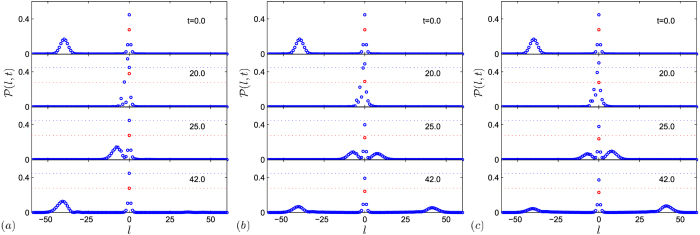
Collision process between an incident photon wave packet and a polariton. The probability distributions 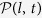 for several instants are obtained by the time evolution under the systems of (**a**) the Hamiltonian *H*_eq_ with *λ* = 2, *U* = 0 and (**b**) *U* = 10, (**c**) the original Hamiltonian *H* with *λ* = 2 (or equivalently, *H*_eq_ with *U* = ∞). The incident wave packet has *k*_0_ = *π*/2 and *α* = 0.3. The blue (red) dotted line indicates the initial probability of the 0th (*e*th) site as comparison. It shows that the probability of the scattering photon is conserved for the non-interacting case with *U* = 0, but not conserved in the presence of nonlinearity in *H*. The result demonstrates the occurrence of stimulated photon emission from a polariton. Moreover, it is observed that the incident wave packet is totally reflected from the center in the case (**a**), but transmitted in the aid of the polariton. The very close similarity between (**b**,**c**) indicates that equivalence between Hubbard and the cavity-atom models. In both cases, the scattered and emitted photons are still local, keeping the similar shape of the incident one.

The probability distribution


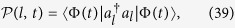


is plotted in Fig. 5 to show the profile of the evolved wave function. One can notice that in the photon-polariton collision process, the probability of the polariton is not conserved. This result has implications in two aspects: First, we achieve a better understanding of the occurrence of stimulated photon emission from a polariton. We find that the scattered and emitted photons are still local. This is crucial for the multi-polariton system, since the outcome photons can stimulate the photon emission of another polariton with high probability. Second, it provides evidence to support the equivalence between *H*_eq_ with large *U* and the original *H*.

**Figure 6:**
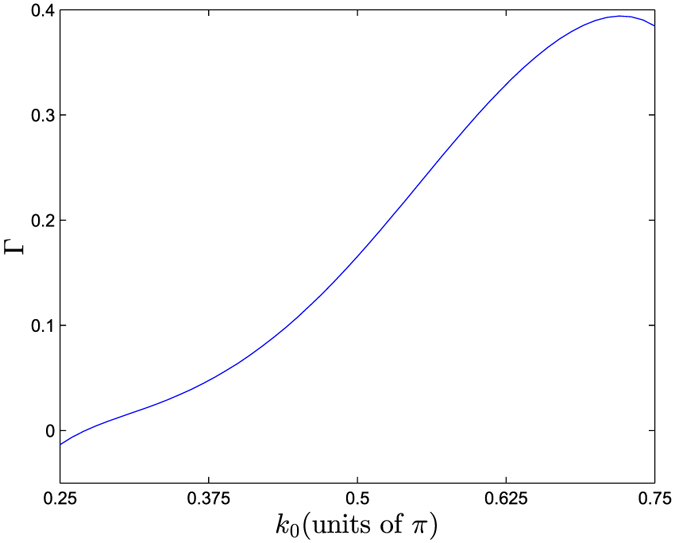
Emission probability from polariton sized *l*_0_ = 9, stimulated by the photon wave packet with *α* = 0.3 and different *k*_0_, for the system with *λ* = 2. It shows that the transition probability can reach 0.4 at 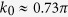.

The above result is for an incident wave packet with *k*_0_ = *π*/2. We are interested in the dependence of emission probability on the central momentum *k*_0_ of the incident wave packet. The probability of the survival polaritons can be measured approximately by the photon probability within the region of the initial polariton resides in, i.e.,


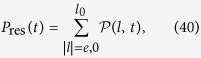


where *l*_0_ denotes the extent of the polariton. Obviously, *P*_res_(*t*) contains the probabilities of the residual polariton and the free photons within 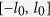. For infinite chain system, 1 − *P*_res_(∞) equals to the photon emission probability Γ. In the numerical simulation, the system is finite, we take Γ = 1 − Min[*P*_res_(*t*)] within a finite time interval in order to avoid the error from the reflected photons. Results of Γ as function of *k*_0_ presented in Fig. 6, show that the maximal photon emission probability reaches 0.4 at 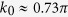. We can see that the stimulated transition is significant, which indicates that a polariton is fragile against an incident photon.

**Figure 7:**
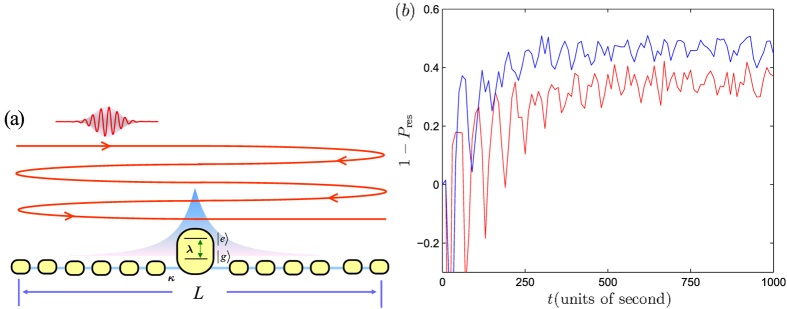
(**a**) Schematic illustration for the scattering process of a moving wave packet and a stationary polariton at center of finite chain. (**b**) Plots of 1 − *P*_res_(*t*) for the cases with *l*_0_ = 9, *α* = 0.3, *λ* = 2, *L* = 120, *k*_0_ = 3*π*/4 (blue), *π*/2 (red). One can see that the probability converges to a nonzero constant at long-time scale.

Now we explore a system with a portion of cavities with doped atom. For a well prepared insulating phase, which is formed by many independent polaritons, decreasing *λ* can lead to the delocalization of the photons. The above analysis offers an alternative probability: external radiation can trigger a sudden change of the state. After the collision of an incident photon and the first polariton, the scattered and emitted photons can further stimulate other polaritons. In order to mimic such a chain reaction, we study the multi-collision process by computing the time evolution of the two-particle system in a long time scale. We consider a finite system, in which the scattered and emitted photons are reflected due to the open boundary condition. It can simulate the repeating collision process, resulting in the continuous probability decay of polaritons. Results of our numerical simulations of 1 − *P*_res_(*t*) is presented in Fig. 7(b). It appears that the local average of *P*_res_(*t*) continuously decays at beginning as predicted and then converges to a nonzero constant. As pointed above, *P*_res_(*t*) may contain photon probability, leading to *P*_res_(*t*) > 1. However, the local maxima of 1 − *P*_res_(*t*) can measure the stimulated transition approximately.

**Figure 8:**
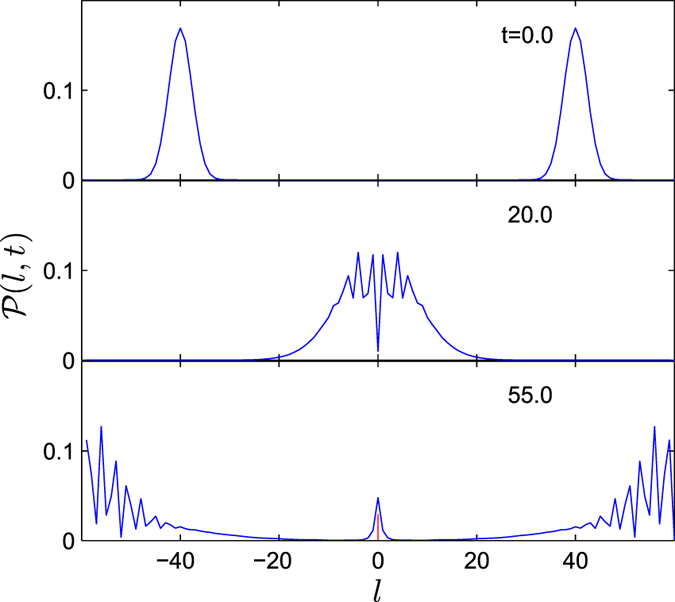
Collision process between two incident photon wave packets from leftmost and rightmost, respectively. The probability distributions 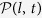 for several instants are obtained by the time evolution under the system of the original Hamiltonian *H* with *λ* = 2 (or equivalently, *H*_eq_ with *U* = ∞). The red line indicates the probability of the *e*th site. It shows that the probability of the scattering photon is not conserved in the presence of nonlinearity in *H*. It demonstrates the polariton can be created by the collision of two free photons.

We presume that a polariton should be washed out by successive collision. However, numerical result shows that the residual polariton probability does not tend to zero after a long time. There are two main reasons: First, as time goes on, any wave packets will spread, reducing the impact of photons on the polariton. Second, the inverse process of photon emission should be considered, in which two colliding photons can create polaritons. To demonstrate such a process, we compute the corresponding simulation. In this process, according to Eq. 36, the initial state can be expressed as


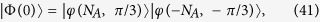


which implies that there are only two symmetry Gaussian wave packets at the beginning. At time *t*, the evolved state is


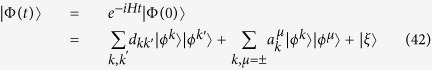


where 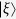 denotes the two-excitation polaritonic state. The probability distribution 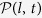 at several typical instants is plotted in Fig. 8. One can see that in the photon-photon collision process, the probability of photons is not conserved as well, which indicates that a polariton can be created when two photons meet at the 0-th cavity. This shows that a polariton can be generated by two-photon Raman scattering. As a summary of numerical results, we conclude that a polariton cannot completely transmit to a photon by the collision from a single photon, and inversely, a photon cannot completely transmit to a polariton by the collision from a single photon. The essential reason is the energy conservation: two-photon energy cannot match that of one photon plus one polariton, i.e.,


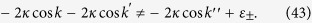


This feature can also be employed to realize all-optical control of photon storage. One main task of quantum information science is to find physical implementations in which a flying qubit can be stopped to store or process quantum information. It has been shown that a flying qubit can be stopped and stored as a collective polariton by tuning the cavity-atom coupling strength adiabatically^27^. In the present cavity QED system, a single-photon wave packet can be a flying qubit, while a polariton can be regarded as a stopped photon, or a stationary qubit. Our result indicates that a single-photon wave packet, or a train of separated wave packets cannot excite a polariton if the atom is in ground state at the beginning. Then any incident single photons from one side cannot create a polariton solely, leaving the atom in the ground state. This can be expressed as equation


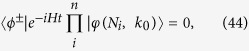


where *N*_*i*_ < 0, *k*_0_ ∈ (0, *π*) and 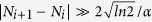, i.e., all the *n* wave packets incident from left and the neighboring wave packets are well separated. In contrast a photon can be stopped at the polariton with the aid of single photons train from the opposite side. This can be expressed as equation


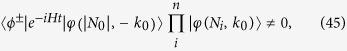


i.e, the atom partially absorbs a photon to form a polariton. The processes expressed by two above Eqs. are schematically illustrated in Fig. 3(b).

## Discussion

In this paper, the scattering problem of photon and polariton in a one-dimensional coupled-cavity system has been theoretically investigated. The analysis shows that, a photon can stimulate the photon emission from a polariton, which suggests that the insulating phase is fragile against the external radiation for a system with a lower density of doped cavity. This result can have some applications in practice. For example, this provides a way to induce the amplification of the photon population in a multi-polariton system as a photon amplifier. On the other hand, we also find that two-photon Raman transition can occur in this cavity QED system, i.e., a stationary single-excitation polariton can be generated by three-body, two photons and atom, collision. This phenomenon can be used to design a scheme to stop and store a single photon. Although this two photon-polariton transitions is probabilistic, it reveals the peculiar features of two-excitation dynamics, which significantly differs from a single-particle scattering problem and opens a possibility to achieve all-optical control of a single photon. The underlying physics can be understood as the effective interaction of two photons arising from the nonlinearity in the doped cavity. These photon emission and absorption processes is an exclusive signature of correlated photons and could be applied to the quantum and optical device design.

## Methods

### The exact eigenstates with 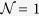


In this section, we present the exact eigenstates with 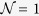 for the Hamiltonian *H*. The Hamiltonian has parity symmetry [*P, H*] = 0, where 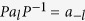. The odd-parity eigenstates can be obtained directly, which is


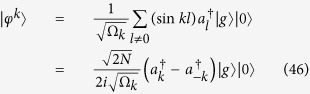


with eigen energy 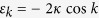, where Ω_*k*_ is the normalization factor and 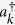 is the photon operator in *k* space, i.e.,


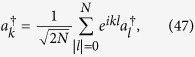



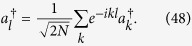


The solutions 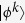 with even parity are two folds:

(i) For real *k*, the eigenstates has the form


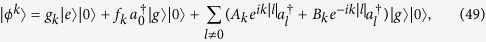


where


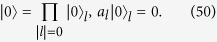


Submitting 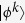 to the Schrodinger equation


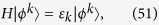


we get the equations for coefficients *g*_*k*_, *f*_*k*_, *A*_*k*_, and *B*_*k*_,


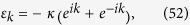



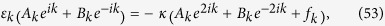



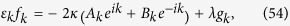



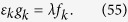


The eigenstates 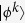 are two folds:

(i) For real *k*, a straightforward derivation leads to


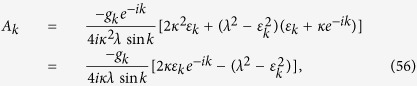



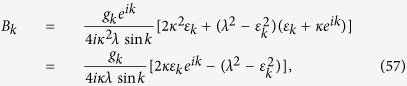



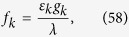



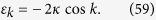


Then we have


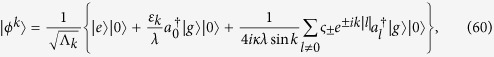



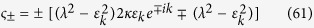


where Λ_*k*_ is the normalization factor, and 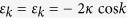. These are extended states.

(ii) There are two eigenstates with complex *k* which can be seen as two bound states. The boundary condition


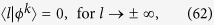


and real *ε*_*k*_ require


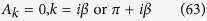


with real *β* > 0. A straightforward derivation leads to


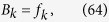



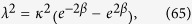



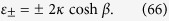



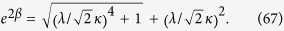


Then we have


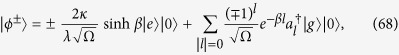


where the normalization factor is


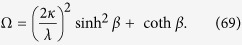


## Additional Information

**How to cite this article**: Li, C. and Song, Z. Stimulated photon emission and two-photon Raman scattering in a coupled-cavity QED system. *Sci. Rep.* **6**, 20991; doi: 10.1038/srep20991 (2016).
